# Transcriptional mechanisms underlying sensitization of peripheral sensory neurons by Granulocyte-/Granulocyte-macrophage colony stimulating factors

**DOI:** 10.1186/1744-8069-9-48

**Published:** 2013-09-25

**Authors:** Kiran Kumar Bali, Varun Venkataramani, Venkata P Satagopam, Pooja Gupta, Reinhard Schneider, Rohini Kuner

**Affiliations:** 1 Institute for Pharmacology and Molecular Medicine Partnership Unit, Heidelberg University, Im Neuenheimer Feld 366, D-69120 Heidelberg, Germany; 2 Luxembourg Centre for Systems Biomedicine (LCSB), University of Luxembourg, Campus Belval, House of Biomedicine, 7 avenue des Hauts-Fourneaux, L-4362 Esch-sur-Alzette, Luxembourg; 3 European Molecular Biology Laboratory, Meyerhofstrasse. 1, D-69117 Heidelberg, Germany

## Abstract

**Background:**

Cancer-associated pain is a major cause of poor quality of life in cancer patients and is frequently resistant to conventional therapy. Recent studies indicate that some hematopoietic growth factors, namely granulocyte macrophage colony stimulating factor (GMCSF) and granulocyte colony stimulating factor (GCSF), are abundantly released in the tumor microenvironment and play a key role in regulating tumor-nerve interactions and tumor-associated pain by activating receptors on dorsal root ganglion (DRG) neurons. Moreover, these hematopoietic factors have been highly implicated in postsurgical pain, inflammatory pain and osteoarthritic pain. However, the molecular mechanisms via which G-/GMCSF bring about nociceptive sensitization and elicit pain are not known.

**Results:**

In order to elucidate G-/GMCSF mediated transcriptional changes in the sensory neurons, we performed a comprehensive, genome-wide analysis of changes in the transcriptome of DRG neurons brought about by exposure to GMCSF or GCSF. We present complete information on regulated genes and validated profiling analyses and report novel regulatory networks and interaction maps revealed by detailed bioinformatics analyses. Amongst these, we validate calpain 2, matrix metalloproteinase 9 (MMP9) and a RhoGTPase Rac1 as well as Tumor necrosis factor alpha (TNFα) as transcriptional targets of G-/GMCSF and demonstrate the importance of MMP9 and Rac1 in GMCSF-induced nociceptor sensitization.

**Conclusion:**

With integrative approach of bioinformatics, *in vivo* pharmacology and behavioral analyses, our results not only indicate that transcriptional control by G-/GMCSF signaling regulates a variety of established pain modulators, but also uncover a large number of novel targets, paving the way for translational analyses in the context of pain disorders.

## Background

Pain is one of the most severe and common symptoms of a variety of cancers and is a primary determinant of the poor quality of life in cancer patients. In a large number of clinical cases, cancer-associated pain, particularly the neuropathic component thereof, is resistant to conventional therapeutics or their application is severely limited owing to the widespread side effects. Because many types of carcinomas and sarcomas metastasize to skeletal bones, they are associated with spontaneous pain, hyperalgesia and allodynia. As potential mechanisms, tumor-derived factors, such as NGF [1], endothelins [2-4], amongst others, have been studied, which either directly activate nociceptive nerves or sensitize them towards sensory stimuli [5,6].

Several types of non-hematopoietic tumors secrete hematopoietic colony stimulating factors, which act on myeloid cells and tumor cells [7]. In a recent study, we demonstrated that receptors and signaling mediators of granulocyte- and granulocyte-macrophage colony stimulating factors (G-/GMCSF) are also broadly expressed on sensory nerves in mouse models of bone metastases as well as in human biopsies of pancreatic adenocarcinoma [8]. Using animal models of bone metastases which closely mimic the nature and progression of cancer pain in humans, we reported that GCSF and GMCSF directly act on receptors on diverse DRG neurons to subserve important functions in the generation of pain hypersensitivity in tumor-affected regions [8]. Importantly, behavioral, electrophysiological and biochemical experiments demonstrated sensitization of sensory nerves towards thermal and mechanical stimuli as well as an increase in neurotransmitter release upon exposure to G-/GMCSF. By adapting RNAi methodology *in vivo*, we demonstrated that a specific loss of GMCSFRα in DRG led to a reduction in bone tumor–evoked pain without interfering with the tumor growth, indicating that GMCSF signaling in peripheral nerves contributes substantially to cancer pain [8]. Recent studies on post-surgical pain and inflammatory pain also point to a key role for these cytokines [9-12].

G-/GMCSF activates the JAK family of receptor tyrosine kinases, which unfolds its activity by not only regulating enzymes and target proteins within its local milieu, but importantly also by activating the STAT family of transcription factors, which subsequently dimerize and translocate to the cell nucleus to regulate gene expression [13]. Albeit we have reported local, acute activation of the ERK Kinase as well as PI3 Kinase in sensory nerves upon a short-term exposure to G-/GMCSF, nothing is known so far about the nature of genes regulated transcriptionally in DRG neurons upon exposure to G/GMCSF. However, long-term transcriptional mechanisms of G/GMCSF action are arguably of even greater importance in pathophysiological states involving chronic, continual release of G/GMCSF, such as tumor-affected tissues, rheumatoid arthritis, amongst others [14,15]. Addressing precise mechanisms via which the G-/GMCSF-JAK-STAT pathway elicits long-term nociceptive sensitization is thus important for understanding mechanisms of cancer pain and other chronic disorders associated with G-/GMCSF release.

In lieu of the attractive therapeutic opportunities offered by these findings, we aimed to elucidate cellular targets of G/GMCSFR in DRG neurons, particularly with respect to transcriptional regulation. Not only did we find a variety of known, established ‘pain-related’ mediators to be transcriptional targets of G-/GMCSF, but also several protein-protein interaction hubs were observed to be under G-/GMCSF regulation in sensory neurons via detailed bioinformatics analyses. Behavioral and pharmacological analyses on 4 of the emerging targets confirmed that Rac1 and Matrix metallopeptidase 9 (MMP9) contribute to GMCSF-induced nociceptive sensitization. These integrative approaches advance our understanding of chronic pain mechanisms and hold promise in the development of novel therapeutic approaches.

## Materials and methods

### Animal usage

All animal usage procedures were in accordance with ethical guidelines laid down by the International Association of the Study of Pain and the local governing body (Regierungspräsidium Karlsruhe). All behavioral measurements were done in awake, unrestrained, age-matched adult (more than 2 months-old) C57/Bl6 mice. Mice were housed in plastic cages, with ambient temperature and a 12 h diurnal light cycle. Food and water were provided *ad libitum*.

### Sensory neuronal cultures and G-/GMCSF treatment

Adult DRG neuronal cultures were prepared following the protocol explained previously [8]. Briefly, neuronal cells isolated from adult wild type mice were seeded on Poly-L-Lysine coated cover slips and maintained in F12 Media (Sigma) supplemented with 15% Amino Acids (Gibco), 10% bovine serum (Invitrogen), 1% Penicillin/Streptomycin (Gibco), 0.5% L-Glutamine (Gibco) and Nerve Growth Factor (100 mg ml-1, Roche). 4 days old enriched adult neuronal cultures were starved of growth factors and serum for 4 h. At the end of 4 h, starving culture medium was replaced with medium containing 0.5% Fetal bovine serum (Life Technologies, 10270106) together with either 1× PBS (vehicle) or 200 ng/mL of murine GMCSF (Peprotech, 315–03) or 200 ng/mL of murine GCSF (Peprotech 250–05) dissolved in 1×PBS. Neurons were left in the incubator for 24 h. Each treatment was performed in triplicate culture wells (n = 3) to test biological variability. At the end of 24 h, total RNA was isolated and used for microarray expression or qRT-PCR analysis.

### RNA isolation from cultured sensory neurons and DRGs

Total RNA from cultured sensory neurons treated with murine GMCSF or GCSF or PBS for 24 h was isolated using mirVana™ miRNA Isolation Kit (Ambion, AM 1561) following manufacturer’s instructions and dissolved in 20 μl of nuclease-free water. Purification steps were performed using RNAse-free DNAse kit (Qiagen, 79254) following manufacturer’s instructions. RNA concentration was determined using the NanoDrop spectrophotometer (NanoDrop Technologies, Wilmington, Germany) and the quality of total RNA was checked by gel analysis using the total RNA Nanochip assay on an Agilent 2100 Bioanalyzer (Agilent Technologies GmbH, Waldbronn, Germany). Only samples with RNA index values greater than 7 were selected for mRNA profiling. 200 ng of total RNA from each biological sample was used as starting material for mRNA expression analysis.

For in vivo testing, lumbar DRGs L3, L4 and L5 were collected at 25 h, 36 and 48 h after bilateral intraplantar application of 20 ng murine GMCSF and flash frozen in liquid nitrogen. Total RNA was isolated and processed following the same protocol explained above for cultured sensory neurons.

### Microarray expression, networking and gene ontology analysis

The mRNA profiling was performed on polyadenylated RNA using Illumina mouse sentrix-6 chips. cDNA library preparation, hybridization and scanning steps were performed by employing in-house standardized protocols and including stringent positive and negative controls at each step at the genomics and proteomics core facility, German Cancer Research Centre (DKFZ, Heidelberg, Germany). The array intensity data were imported into Beadstudio ver. 3 from Illumina and the quantile array normalization method was employed to account for intra- and inter-array variations in expression intensities within each experimental group [16]. Magnitude of induction or repression of individual transcript was compared over vehicle-treated samples. To be able to understand the magnitude of regulation at transcript level, we first converted probe-level signals to transcript-level signals by using in house developed Perl scripts and applying following criteria – i) Take the average fold-change if all the probes for one transcript showed the same direction (positive or negative) of regulation, ii) Discard the transcripts for which different probes showed different directions of regulation iii) Take the regulation value from the majority of probes if only one probe out of several probes is showing different expression signals.

All the networking analyses of the expression data were performed using MetaCore™ software in which a network is built around an initial list of “seed nodes,” which can originate from the uploaded experiment, or be manually assembled, or else be automatically converted by MetaCore™ from a list of genes. For the gene ontology enrichment analyses, we used software called bioCompendium (http://biocompendium.embl.de) developed at the European Molecular Biology Laboratory, Heidelberg, Germany.

### GMCSF and inhibitors application *in vivo*

Murine GMCSF was purchased from Peprotech, dissolved in 1× PBS of physiological PH and 20 ng was applied into the intraplantar surface of adult C57/Bl6 mice unilaterally for 4 times at 8 h intervals. Inhibitors for MMP-9 (CAS 1177749-58-4) and Rac1 (1090893-12-1) were purchased from Calbiochem and dissolved in 10% and 50% Dimethyl Sulfoxide (DMSO, Applichem), respectively. Calpain 1/2 inhibitor (Calpain inhibitor III, A3672, 0250) was purchased from Sigma-Aldrich and dissolved in 20% DMSO. TNFα inhibitor was purchased from Pfizer (Enbrel®^)^) and diluted in 1× PBS. One hour after the last GMCSF dosage application, different groups of mice received 0.15 pmoles, 1.5 nmoles, 10 nmoles or 100 pmoles of MMP-9 or Rac1 or Calpain 1/2 or TNFα inhibitors, respectively, in 10 μl volume of vehicle into the same paw into which multiple dosages of GMCSF were applied. BSA dissolved in 1× PBS was used as vehicle control for TNFα inhibition experiments. Mechanical hyperalgesia was recorded after 4 and 8 h after the last GMCSF dosage application while thermal hypersensitivity was recorded after 5 and 9 h of the last GMCSF dosage application.

### Mechanical and thermal pain behavioral tests

Mice were habituated to the experimental setup in at least 2 separate sessions within the week preceding the time of behavioral testing. The observer was fully blinded to the identity of the groups in all behavioral tests. To measure mechanical sensitivity, animals were placed on an elevated wire grid and the plantar hind paw was stimulated using calibrated von Frey monofilaments of 0.07 g, 0.16 g, 0.4 g and 1.0 g strength (Bioseb, France). Paw withdrawal was recorded as a positive response. Data is expressed as percentage of frequency of response over 5 stimulations and data from representative filament is shown in this manuscript. For thermal nociceptive testing, radiant heat was applied using Hargreaves’ apparatus (Ugo Basile, Italy) to the plantar surface of the hind paw, until mice retract it sharply. The time taken to retract (paw withdrawal latency) the hind paw was recorded. A cut-off of 15 seconds heat exposure was followed in order to avoid any potential damage to the tissue.

### Quantification of mRNA expression

We used NanoString-nCounter™ based gene quantification method to validate microarray expression data. Probes specifically targeting the desired gene of interest were obtained from Nanostring Technologies, USA and analyses were performed at the nCounter core facility of the Medical Faculty of Heidelberg, Heidelberg University, Germany. Two hundred ng of total RNA were used to analyze the expression of diverse target genes, using 5 housekeeping genes, namely Clathrin, heavy polypeptide (*Cltc*), Glyceraldehyde-3-phosphate dehydrogenase (*Gapdh*), glucuronidase beta (*Gusb*), Hypoxanthine guanine phosphoribosyl transferase (*Hprt*) and Tubulin, beta 5 class I (*Tubb5*), as internal controls. Expression of target genes was analyzed by comparing treated and control samples. Fold-change of test gene was expressed as arithmetic average value over all 5 housekeeping genes.

Taqman assays (Life Technologies, USA) were used for QRTPCR-based quantification of Rac1 (assay ID Mm01201653_mH), Calpain2 (assay ID Mm00486669_m1), MMP9 (assay ID Mm00600164_g1) and TNFα (assay ID Mm00443260_g1). 20 ng of total RNA was used to prepare the cDNA using random primers from the High Capacity cDNA Reverse Transcription Kit (Applied Biosystems, 4368814) following manufacturer’s instructions. Four μl of prepared cDNA were PCR amplified in each reaction using mRNA-specific primers and TaqMan® Universal Master Mix II, (Applied Biosystems, 4440040) following manufacturer’s instructions on Chromo 4 detection system (BioRad, USA). The expression level of the target mRNA was normalized to the expression of Glyceraldehyde 3-phosphate dehydrogenase (GAPDH, assay ID 4352932E, Applied biosystems). Each mRNA was amplified from triplicate samples and Ct values were recorded. Fold-change in the mRNA expression in vehicle- or GMCSF-treated sensory neuronal cultures was calculated using *D*D*C*T method [17] which measures the relative change in expression of a mRNA from treatment to control compared to the reference gene.

### Data analysis

All data are presented as mean ± standard error of the mean (S.E.M.), Two-tailed Student’s *t*-test or the Analysis of Variance (ANOVA) for repeated measures followed by post-hoc Fisher’s LSD test was utilized to determine statistically significant differences (p < 0.05), unless mentioned otherwise for a particular experiment.

## Results

### GMCSF-mediated changes in the gene expression repertoire in sensory neurons

To investigate transcriptional expression changes caused by exposure to GMCSF or GCSF application at a genome-wide level, we performed a genome-wide gene profiling screen from cultured DRG neurons derived from adult mice. Neuron-enriched cultures were starved of growth factors and serum for 4 h and treated with GMCSF or GCSF (200 ng/mL in PBS) or vehicle (PBS) in medium containing 0.5% serum for 24 h. Total RNA isolated from 3 such independent experiments was subjected to quality control as described under methods and processed in profiling experiments using Illumina prespotted arrays. cDNA library preparation, hybridization and scanning steps were performed by employing in-house standardized protocols, and including stringent positive and negative controls at each step. The array intensity data were imported into Beadstudio ver. 3 from Illumina and the quantile array normalization method was employed to correct for systematic differences between arrays which do not represent a biological variation of interest between experimental groups [18]. Magnitude of fold-induction or fold-repression of individual transcript was compared over vehicle-treated samples, as described in our previous studies [19].

**Figure 1:**
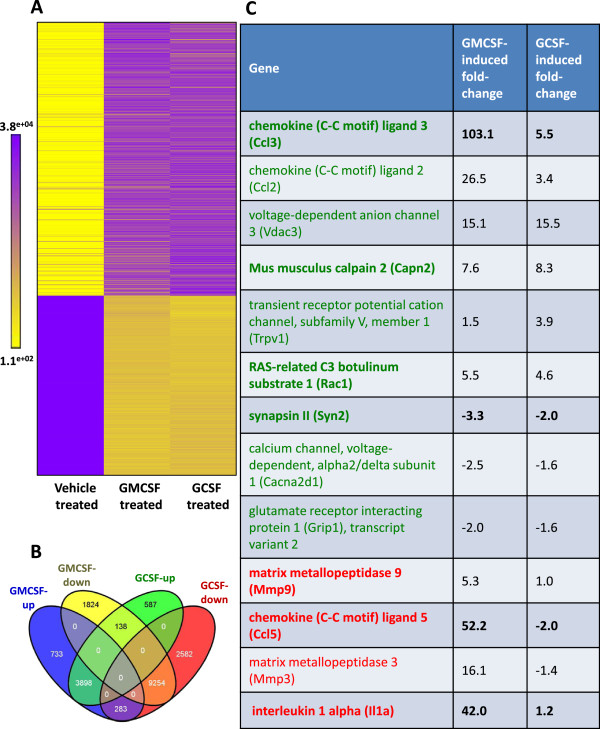
**GMCSF- or GCSF- mediated gene pool in the peripheral sensory neurons. (A)** Heat map representation of significantly-regulated transcripts showing more than two-fold change (two-tailed *t*-test assuming equal variance, P with Benjamini and Hochberg False Discovery Rate correction <0.05) following chronic exposure to GMCSF or GCSF in sensory neurons of the DRG. The color scale represents quantile normalized hybridization intensities for each gene. **(B)** Venn diagram representing the number of genes commonly or differentially regulated by GMCSF and GCSF exposure in DRG neurons. **(C)** Selected pain-related genes that were significantly regulated (two-tailed *t*-test assuming equal variance, P with Benjamini and Hochberg False Discovery Rate correction <0.05). Genes commonly (green text) or differently (red text) regulated by GMCSF and GCSF stimulation in sensory neurons are shown. Gene regulation confirmed by qRT-PCR analysis is highlighted in bold text.

Data acquired from the array provided expression signals from 46090 probes targeting 30723 genome-wide transcripts. Out of 30723 transcripts arrayed 15833 and 16882 showed significant fold change in their expression in GMCSF-treated and GCSF-treated samples, respectively, as compared to vehicle-treated samples (P ≤ 0.05, two-tailed *t*-test assuming equal variance, Benjamini and Hochberg false discovery rate correction, n = 3 independent profiling experiments) (Figure 1 and Additional file 1: Table S1). Because both GMCSF and GCSF act in a pronociceptive manner, we then studied commonly regulated transcripts and found that 3898 transcripts showed significant upregulation with GMCSF as well as with GCSF as stimuli for DRG neurons (Figure 1B). These included several genes which have been implicated in nociceptive modulation, such as chemokine (C-C motif) ligand 2 and 3 (*Ccl2 and Ccl3)*[20,21], transient receptor potential cation channel, subfamily V, member 1 (*TRPV1*), a molecular sensor and transducer for heat, protons and algogens [22], amongst others (a few examples are shown in Figure 1C). Moreover, 9254 transcripts were commonly downregulated upon exposure with GMCSF as well as with GCSF. These also included several pain-related known genes, such as the voltage-dependent calcium channel subunit aplha2/delta1 (*Cacna2d1*) [23], the AMPA receptor interacting protein, GRIP1 [24,25], amongst others (Figure 1C). Interestingly, however, 421 genes showed reciprocal regulation upon exposure to GMCSF or GCSF (Figure 1B), e.g. the nociceptive modulatory chemokine (C-C motif) ligand (*Ccl5*) [26] and the matrix metalloprotease 3 (*MMP3*) [27,28] were significantly upregulated after GMCSF exposure, but downregulated upon exposure to GCSF (Figure 1C). In general, genes encoding nociceptive modulating pronociceptive chemokines and cytokines appeared more strongly regulated by GMCSF signaling than by GCSF signaling in DRG neurons (some examples in Figure 1C).

**Figure 2:**
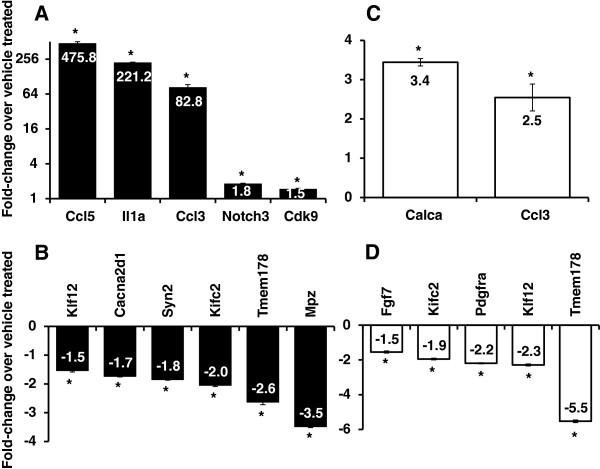
**Nanostring-nCounter-based validation of selected genes those are up-regulated by GMCSF (A), down-regulated by GMCSF (B), up-regulated by GCSF (C) and down-regulated by GCSF (D) in the sensory neurons.** Fold-change expression in the genes is expressed as arithmetic average over 5 housekeeping genes namely *Cltc, Gapdh*, *Gusb*, *Hprt* and *Tubb5* in all panels. * P < 0.05, one-way ANOVA followed by Fisher’s LSD Post-hoc analysis.

To test the validity of the microarray data reported above, we performed quantitative measurements of the expression of several candidate regulated genes using Nanostring-nCounter technique. Amongst putatively regulated transcripts in the GMCSF-mediated gene pool, we tested 100 genes quantitatively and found that 78 genes were regulated as predicted by profiling data, which included up-regulated genes such as chemokine (C-C motif) ligand 5 (*Ccl5*), interleukin 1 alpha *(Il1a), Ccl3* (Figure 2A) and down-regulated genes such as *Cacna2d1,* synapsin II (*Syn2*), amongst others (Figure 2B). Along the same lines, GCSF-mediated regulation of several genes, including pain-related genes such as calcitonin/calcitonin-related polypeptide, alpha, transcript variant 1(*Calca*), *Ccl3*, and fibroblast growth factor 7 (*Fgf7*) amongst several others could be confirmed using the same PCR-based methodology (Figure 2C, 2D). These results thus validate the results obtained with the microarray expression arrays via an independent method.

**Figure 3:**
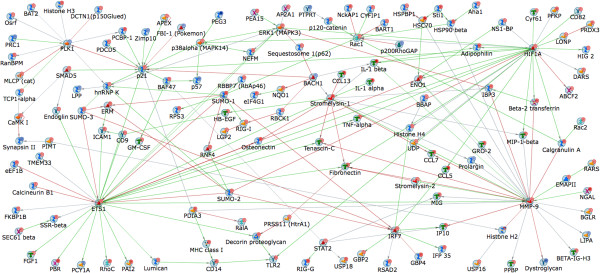
**Direct-interactions-network analysis on GMCSF-mediated gene pool with fold-change between +4 and −4 as compared to control-treated sensory neurons and P-BH < 0.05 (*t*-test, P with Benjamini and Hochberg False Discovery Rate < 0.05).** Genes upregulated and downregulated in GMCSF-dependent manner are marked with red and blue circles, respectively. Please see Additional file 3: Figure S2 for information on legends.

**Figure 4:**
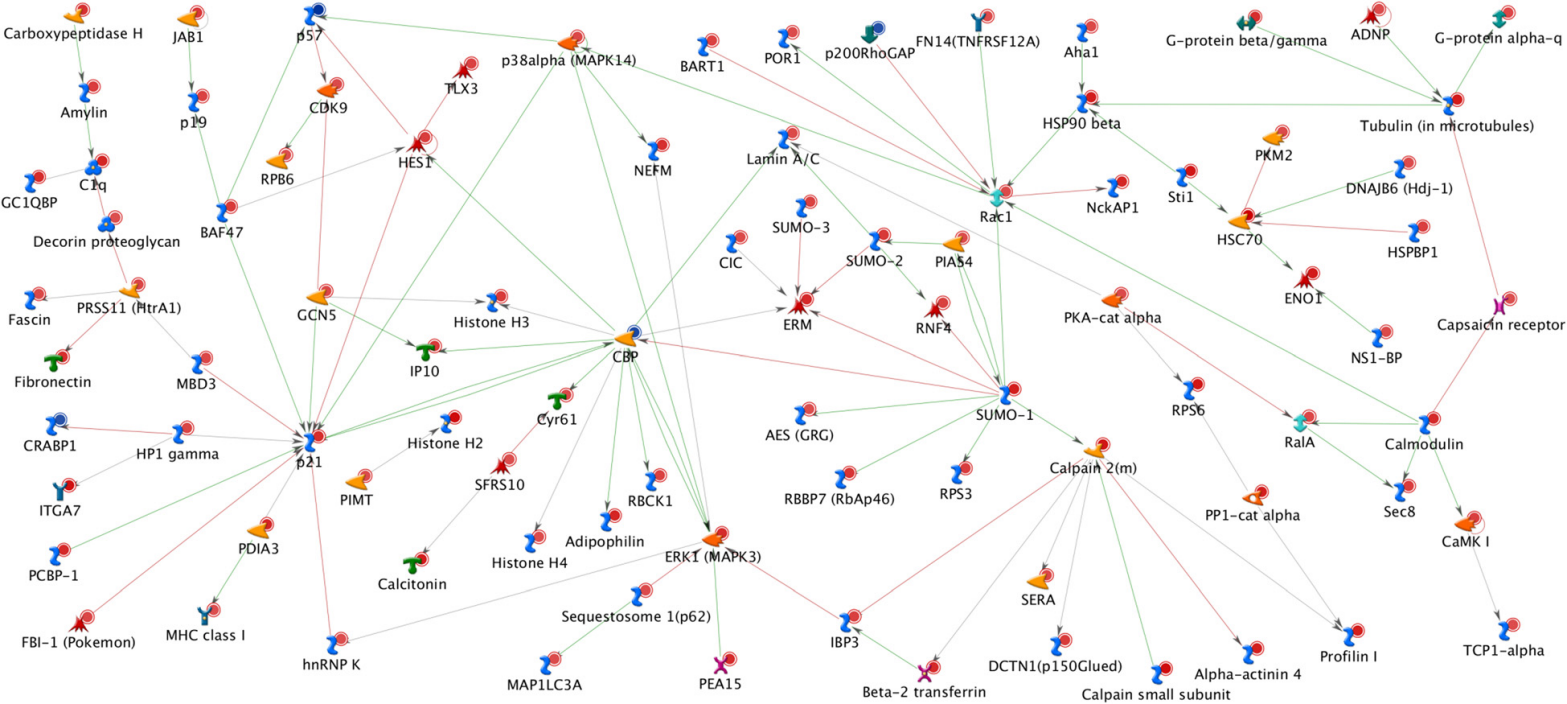
**Direct-interactions-network analysis on GCSF-mediated gene pool with fold change between +4 and −4 and as compared to control-treated sensory neurons and P-BH < 0.05 (*t*-test, P with Benjamini and Hochberg False Discovery Rate < 0.05).** Genes upregulated and downregulated in GCSF-dependent manner are marked with red and blue circles, respectively. Please see Additional file 3: Figure S2 for information on legends.

In a next step, to understand systems level interactions in the GMCSF- or GCSF-mediated gene pools, we performed a direct-interactions analysis using Metacore software [19,29]. When we applied this to all significantly regulated transcripts following the criteria explained above for Figure 1, it yielded too dense a network to allow meaningful interpretations (data not shown). Therefore, we stringently filtered out the transcripts which showed at least 4-fold up- or down-regulation upon exposure to GMCSF (thus arriving at 661 transcripts) or GCSF (611 transcripts). Of these, only 467 GMCSF-target genes and 454 GCSF-target genes were well annotated with known higher level mapping in Metacore and were used for the direct-network analysis. The network map generated by the genego direct-interaction network analysis tool revealed a dense network of genes in the GMCSF-target pool with 3 major nodal points namely, two transcription factors, E26 avian leukemia oncogene 1, 5' domain, transcript variant 2 (*Ets1*), Hypoxia inducible factor 1 alpha subunit (*Hif1a*) and a metallo-protease, namely *Mmp9* (Figure 3). These 3 nodal points are intensively related to many kinases such as mitogen-activated protein kinase 3 *(MAPK3),* generic binding proteins such as *Synapsin,* Ras super family members such as *Rac1,* receptors like Toll-like receptor 2 encoding gene *(Tlr2*)*,* all of which are either directly or indirectly implicated in nociceptive mechanisms. Similarly, the direct-interaction network for the GCSF-mediated gene pool also revealed a densely connected network with genes encoding the key posttranslational sumoylation protein (*Sumo1*), the cyclin-dependent kinase inhibitor 1A (*Cdkn1a*), CREB binding protein (*Crebbp or CBP*), calpain 2 (Capn2), *MAPK3* and the RhoGTPase Rac1 (*Rac1*) serving as major nodal points. These nodes are intensively linked to genes encoding Calmodulin 2 (*Calm2*), the Transient Receptor Family channel V1 (*Trpv1 or capsaicin receptor*), Actin-modulatory protein profilin 1 (*Pfn1*), among several others (Figure 4). These results indicate that GMCSF- and GCSF-signaling interlinks transcriptional and post-translational modification mechanisms to key nociceptive modulatory proteins. We further performed direct interaction network analysis on genes that were commonly regulated following GMCSF or GCSF exposure. These commonly-regulated networks revealed shared nodal points such as Rac1, mitogen-activated protein kinase 3 (*Mapk3*), among others (Additional file 2: Figure S1 and Additional file 3: Figure S2).

A gene ontology enrichment analysis on the same subsets of GMCSF- or GCSF-target genes performed using the bioCompendium software revealed that a major proportion of transcripts (> 50%) show protein-binding activity. Interestingly, cytokine activity and chemokine-receptor binding categories were found to be represented in the molecular function enrichment analysis on GMCSF-target pool in DRG neurons (Additional file 4: Figure S3), consistent with our observation of high regulation levels of several nociception-related cytokines and chemokines.

In the next step, by using the same subsets of significantly regulated GMCSF- or GCSF-modulated genes as explained for Figure 1A, we performed a network analysis in which networks are built on the basis of relationships and interactions contained in the MetaCore™ Database. Interestingly, the network which emerged with a top rank from the gene pool of GMCSF-targets in sensory neurons revealed that the classical signaling cascade consisting of JAK kinases (JAK1 and JAK2) and STAT transcription factors, STAT1 and STAT3 are tightly linked to Tumor necrosis factor-alpha (TNF-alpha) and its receptor TNF-R1, both of which were observed to be directly regulated by GMCSF in sensory neurons in our profiling analyses (Additional file 5: Figure S4). Moreover, a link to NF-kappa-I Kappa B signaling, which has also been implicated in sensory neurons [30], was also apparent (Additional file 5: Figure S4). These findings further indicate a close link between GMCSF-induced transcriptional control and induction of key nociceptive modulators, such as TNF-alpha.

### Functional significance of GM-/GCSF-regulated gene pool in GM-/GCSF-induced nociceptive hypersensitivity

**Figure 5:**
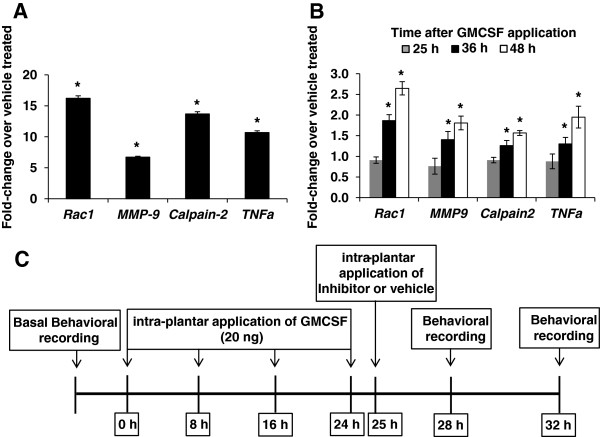
**QRTPCR-based validation of GM-CSF-induced regulation of the expression of *Rac1, MMP9, Calpain2 and TNFα in vitro *as well as *in vivo*.** Gene regulation at 24 h following the start of GMCSF exposure in cultured sensory neurons is shown in **(A)** and in DRGs at different time points following intraplantar GMCSF injection is shown in **(B)**. * denotes P ≤ 0.05 as compared to vehicle group, One-Way ANOVA followed by Fisher’s LSD Post-hoc analysis, n = 3 mice per group. **(C)** Schematic representation of the protocol followed to investigate changes in GMCSF-mediated mechanical and thermal hypersensitivity upon inhibition of Rac1 or MMP9 or Calpain 2 or TNFα *in vivo*.

Finally, to functionally validate our results on GMCSF- and GCSF-associated genes, we selected protein products of a set of four candidate genes from different functional classes and with functional relevance to pain modulation, namely the RhoGTPase Rac1, the matrix metallopeptidase 9 (MMP9), a chemokine TNF-alpha and a generic protease calpain 2*.* To confirm GMCSF-mediated modulation of these four genes, we compared their mRNA expression in the total RNA isolated from the DRG neuronal cultures following chronic treatment with GMCSF or vehicle, i.e. a similar paradigm as with the expression array screening. Analysis of results confirmed GMCSF-mediated robust upregulation of Rac1, MMP9, TNFα and Calpain 2 as compared to vehicle-treated samples (Figure 5A, *P < 0.05, one-way ANOVA followed by Fisher’s LSD Post-hoc analysis).

In previous studies, we have analyzed short-term effects of acute exposure to GCSF and GMCSF [8]. However, in order to mimic chronic clinical conditions which are associated with longer exposure to G-/GMCSF and to match the course of the following behavioral experiments with the time frame of our gene regulation studies, we administered multiple dosages of 20 ng murine GMCSF as described in the scheme shown in Figure 5C and inhibitors were applied one hour after the last GMCSF dosage application. Mechanical sensitivity was recorded upon ipsilateral plantar application of graded von Frey filaments at 3 h and 7 h post injection and thermal sensitivity was recorded using Hargreaves’ apparatus at 4 and 8 h following inhibitor application. For *in vivo* confirmation of GMCSF-mediated regulation of *Rac1, MMP9, Calpain2* and *TNFα*, DRGs were collected at 24, 36 and 48 h after the first GMCSF dosage application. QRTPCR-based expression analysis confirmed GMCSF-mediated up-regulation of Rac1, MMP9, Calpain 2 and TNFα in the DRGs of GMCSF-treated mice as compared to the vehicle-treated group of mice (Figure 5B, *P < 0.05, one-way ANOVA followed by Fisher’s LSD Post-hoc analysis).

### Peripheral Rac1 activation is required for GMCSF-mediated mechanical and thermal hyperalgesia

**Figure 6:**
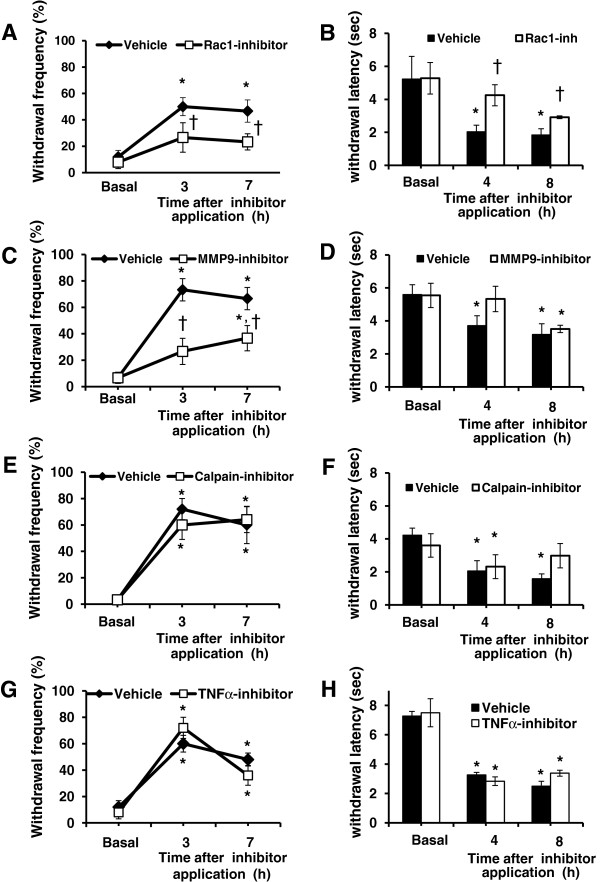
***In vivo *validation of GM-CSF transcriptional targets in DRG neurons in the context of nociceptive sensitization evoked by long-term exposure to GMCSF.** Changes in GMCSF-mediated mechanical hypersensitivity upon inhibition of Rac1 **(A)**, MMP9 **(C)**, Calpain-2 **(E)** or TNFα **(G)** as compared to corresponding vehicle-treated mice are shown. Response frequency to the von Frey filament at 0.16 g force is represented on the Y-axis. Changes in GM-CSF-mediated thermal hypersensitivity upon inhibition Rac1 **(B)**, MMP9 **(D)**, Calpain-2 **(F)** or TNFα **(H)** as compared to corresponding vehicle-treated mice are shown. Withdrawal latency in seconds to calibrated radiant heat is represented on the Y-axis. * denotes P ≤ 0.05 as compared to basal values, ^†^ denotes P ≤ 0.05 relative to corresponding vehicle treated group, One-Way ANOVA with repeated measures followed Fisher’s LSD Post-hoc analysis , n = 6 mice per group.

In our profiling analysis and quantitative PCR analysis, *Rac1* expression increased significantly in DRG neurons following a 24 h-long exposure to GMCSF or GCSF. In spinal neurons, regulation of Rac1 activity is known to impact dendritic spine morphology and density as well as pain hypersensitivity following spinal cord injury [31]. However, Rac1 has not been addressed in peripheral sensory neurons in the context of nociceptive modulation in sensory neurons. To address whether Rac1 is upregulated at 24 h after exposure to GMCSF contributes to GMCSF-evoked nociceptive hypersensitivity, we selected dosage of the Rac1-specific inhibitor, NSC23766 [32], based on the concentration used by Tan et. al [31] in rats. Extrapolating this concentration to mice and to account for the dilution factor in the CSF, we selected approx. 10 times lesser concentration (1.5 nmoles) in the current study. We divided mice treated with GMCSF over 24 h into 2 groups – one received a single intraplantar injection of NSC23766, a specific Rac1 inhibitor (1.5 nmoles in 10 μl of 50% DMSO) and the other group received a single intraplantar injection of vehicle (10 μl of 50% DMSO) 1 h after the last plantar treatment with GMCSF; GMCSF-mediated mechanical and thermal hypersensitivity was analyzed approx. 3 h and 7 h after inhibitor or vehicle application in both groups. Whereas mice injected with vehicle showed significant mechanical hypersensitivity to 0.16 g of von Frey force as compared to vehicle at 3 h as well as 7 h after the inhibitor application (* P = 0.003 at 3 h and 0.007 at 7 h, One-Way ANOVA with repeated measures followed by Fisher’s LSD Post-hoc analysis, n = 6 mice per group), mice injected with the Rac1 inhibitor did not show any significant deviation from basal response frequencies to 0.16 g force (P > 0.05 as compared to basal; n = 6 mice; † P < 0.01 between groups, One-Way ANOVA with repeated measures followed by Fisher’s LSD Post-hoc analysis, Figure 6A and Additional file 6: Table S2). Along the same lines, vehicle-treated mice showed a significant decrease in withdrawal response latencies to plantar heat (i.e. thermal hyperalgesia) as compared to basal values (* P = 0.003 and 0.002 at 4 and 8 h post inhibitor application, respectively, One-way ANOVA followed by Fisher’s LSD Post-hoc analysis, Figure 6B and Additional file 7: Table S3). In contrast, Rac1-inhibitor-treated mice did not show thermal hyperalgesia at 4 h after inhibitor application; furthermore, thermal hyperalgesia at both time points tested after inhibitor treatment was significantly reduced as compared to vehicle-treated mice († P = 0.033 at 4 h and P = 0.019 at 8 h between vehicle- and Rac1-inhibitor-treated mice, One-way ANOVA with repeated measures followed by Fisher’s LSD Post-hoc analysis, Figure 6B Additional file 7: Table S3). As a control to rule out systemic effects of Rac1 inhibitor, we injected inhibitor or vehicle into the paw contralateral to the paw injected with GMCSF – this treatment failed to block GMCSF-induced mechanical and thermal hyperalgesia in the paw ipsilateral to the GMCSF-injected paw (Additional file 8: Figure S5 panels A and C, *P < 0.05 following GMCSF administration, One-Way ANOVA with repeated measures followed by Fisher’s LSD Post-hoc analysis, n = 6 mice). In the same paradigm, mechanical or thermal response frequencies were unaltered as compared to the basal readings in the paw contralateral to the GMCSF-injected paw (Additional file 8: Figure S5 panels B and G, P > 0.05). Similarly, injection of the same dosage of Rac1 inhibitor unilaterally into the intraplantar surface in the absence of GMCSF treatment did not affect mechanical and thermal response frequencies when tested up to 7 h post-injection (Additional file 9: Figure S6, panels A and B). Thus, these results indicate that locally activated Rac1 specifically contributes to both mechanical and thermal hypersensitivity induced by a prolonged peripheral exposure to GMCSF.

### Peripheral MMP9 activation is required for ongoing nociceptive sensitization

In the current study, expression *of Mmp9,* the gene encoding the extracellular matrix protease MMP9, increased by about 5-fold in DRG following GMCSF stimulation, but not following GCSF stimulation. Given that MMP9 has been shown to participate in inflammatory as well as neuropathic pain in a peripheral as well as a spinal context [33-35], we were interested in addressing whether GMCSF-induced upregulation of MMP9 was functionally linked to GMCSF-evoked exaggeration of mechanical and thermal sensitivity. Using the scheme shown in Figure 5C and described in detail above for the Rac1-associated experiments, we administered a single dose of 0.15 pmoles of a potent MMP9 inhibitor in 10 μl of 10% DMSO (vehicle) to the plantar surface 1 h after the last plantar administration of GMCSF. We selected the MMP9 inhibitor dosage based on its high potency (IC50 = 5nM) and its reported intrathecal dosage to attenuate CFA-mediated mechanical allodynia in rats [36]. Upon peripheral MMP9 inhibition, we observed a complete abrogation of GMCSF-evoked mechanical hypersensitivity to 0.16 g of von Frey force as well as thermal hyperalgesia at 3–4 h after MMP9 inhibitor application (Figure 6C and Additional file 6: Table S2 *P < 0.05 as compared to basal values; † P < 0.05 as compared between inhibitor and vehicle groups; One-way ANOVA with repeated measures followed by Fisher’s LSD Post-hoc analysis). This effect on mechanical and thermal hyperalgesia was partially or fully lost, respectively, at 7–8 h after inhibitor application, reflecting the duration of action of a single dose of the MMP9-inhibitor at the low dose utilized in this study (Figure 6D and Additional file 7: Table S3). Similar to the experiments described above with Rac1 inhibition, we observed that injection of the MMP9 inhibitor in the paw contralateral to the paw injected with GMCSF did not significantly influence GMCSF-mediated mechanical and thermal hyperalgesia in the paw ipsilateral to the GMCSF-injected paw (Additional file 8: Figure S5 panels B and D, *P < 0.05 following GM-CSF administration as compared to basal values, One-way ANOVA with repeated measures followed by Fisher’s LSD Post-hoc analysis) nor induced hyperalgesia in the paw contralateral to the GMCSF-injected paw (Additional file 8: Figure S5, panels F and H, P > 0.05). Furthermore, injection of the MMP9 inhibitor in the absence of GM-CSF did not affect nociceptive sensitivity (Additional file 9: Figure S6 panels C and D, P > 0.05 as compared to basal values, One-way ANOVA with repeated measures followed by Fisher’s LSD Post-hoc analysis). These results indicate that similar to Rac1, peripheral MMP9 activation is important for ongoing nociceptive sensitization that develops upon a prolonged exposure to GMCSF.

### Peripheral calpain2 and TNFα are dispensable in the mediation of GMCSF-mediated hyperalgesia

In the current study, *Capn2*, which encodes the calcium-dependent cysteine proteases calpain 2, emerged as a gene showing a high level of induction, common to both GMCSF and GCSF, in sensory neurons. Drugs which inhibit both Calpain 1 and Calpain 2 have been shown to inhibit mechanical hyperalgesia following inflammation or in response to some mediators [37,38]. Therefore, we also tested the potential of a peripherally administered Calpain 1/2 inhibitor (Calpain inhibitor III, 10 nmol in 10 μl of 10% DMSO) to modulate GMCSF-mediated mechanical and thermal hyperalgesia using the paradigm described above and inhibitor concentrations that have been shown to be effective in previous studies on inflammatory pain [37,38]. Our analysis showed, however, that GMCSF-induced hypersensitivity was not significantly different between mice receiving Calpain1/2 inhibitor or vehicle (10 μl of 10% DMSO) in all tests and at all time points tested (Figure 6E and F; Additional file 6: Table S2 and Additional file 7: Table S3; * P < 0.05 as compared to basal values; One-way ANOVA with repeated measures followed by Fisher’s LSD Post-hoc analysis).

In the current study, tumor necrosis factor alpha (TNFα) was upregulated by 10-fold following GMCSF stimulation but only moderately following GCSF stimulation in microarray or QRTPCR experiments. TNFα inhibition in the spinal cord has been reported to be protective against neuropathic pain [39-41] and systemically-applied TNFα inhibitor protects mice from tumor-induced thermal hyperalgesia [42]. Moreover, a specific TNFα inhibitor, namely Etanercept (trade name Enbrel®), has been suggested to be efficacious against autoimmune diseases such as Crohn’s disease [43] and is in clinical practice for the treatment of several peripheral inflammatory diseases such as rheumatoid arthritis [44]. Therefore, we sought out to investigate the potential of peripherally applied TNFα decoy receptor, Etanercept, choosing a dose of 100 pmol, which is slightly higher than the dose reported to reduce neuropathic hypersensitivity [40]. We observed that GMCSF-induced mechanical and thermal hyperalgesia were not significantly different between groups treated with vehicle (BSA) or Etanercept (Figure 6G and H, Additional file 6: Table S2 and Additional file 7: Table S3; * P < 0.05 following GM-CSF administration as compared to basal values, One-way ANOVA with repeated measures followed by Fisher’s LSD Post-hoc analysis). Thus, albeit TNFα is upregulated in peripheral sensory neurons following GM-CSF administration, it does not appear to directly contribute to GM-CSF-induced nociceptive hypersensitivity.

## Discussion

We and others have previously demonstrated that the hematopoietic growth factors, GMCSF and GCSF, sensitize sensory nerves directly via activation of receptors located on sensory nerves and contribute significantly to cancer-associated pain [8]. Several recent studies have extended these results to acute and chronic pain in the context of post-operative pain, inflammatory pain and osteoarthritic pain [9-12]. Thus, understanding the genomic program induced in sensory neurons by this set of key cytokines is crucial for gaining insights into the pathophysiological role of G-/GMCSF, as well as in developing therapeutic options. To understand the molecular mediators via which the GMCSF receptor is exerting its protective pronociceptive affects, we performed a genome-wide expression screen in cultured sensory neurons following G-/GMCSF ligand treatments. Using in-depth systems level *in silico* analysis, and *in vivo* pharmacology to functionally test selected candidate genes, we present G-/GMCSF-mediated transcriptome change and its importance in modulating GMCSF-mediated hyperalgesia in the sensory neurons.

One of the most interesting observations of the present study was that many chemokines such as *Ccl3, Ccl5*, insulin-like growth factor 1 (*Igf1*)*, Vascular endothelial growth factor A (Vegfa), transcript variant 2* and *TNFα*, which are known to play a significant role in pain modulation [20,26,45,46] are regulated in a G-/GMCSF dependent manner in sensory neurons. It was previously reported that the lack of CCL5 results in decreased hypersensitivity in partial sciatic nerve ligation model of neuropathic pain. Further, infiltration of pro-inflammatory cytokines such as tumor necrosis factor-α, [is this supposed to be alpha?] interleukin [IL]-1b, IL-6, and interferon-c was significantly reduced in the damaged nerves while that of anti-inflammatory cytokines such as *IL-4 and IL-10* was significantly increased in the injured nerves following the global CCL5 loss in mice [26]. TNF*α* was shown to have a significant contribution in the development of pain sensitivity following peripheral nerve injury.

Rac1 is a small GTPase ubiquitously expressed in neurons and other cell types. Spinal cord neurons, astrocytes and oligodendrocytes express high levels of *Rac1* mRNA and its expression is elevated following spinal cord injury (SCI) in rats [47]. Intrathecally applied specific Rac1 inhibitor ameliorates SCI-induced changes in spine morphology, attenuates injury-induced hyper excitability of wide-dynamic range neurons, and increases SCI-mediated pain thresholds [31]. Rac1 activity regulates dendritic spine morphology in hippocampal neurons through actin cytoskeleton reorganization and promotes clustering of glutamate AMPA receptors in spines [48-50]. In the current study, *Rac1* expression increased by about 4–5 fold in sensory neurons following exposure to GMCSF or GCSF stimulation, suggesting that this may work as a shared mechanism between GMCSF and GCSF stimuli to modulate functional as well as structural changes in sensory neurons. Indeed, we have previously shown that knocking down GMCSF receptor-alpha in sensory neurons of the DRG attenuates tumor-induced structural remodeling and sprouting of peripheral sensory terminals [8]. Further, inhibiting Rac1 activity in the peripheral terminals resulted in complete protection from GMCSF-induced mechanical hypersensitivity and partial protection from thermal hypersensitivity. These effects of peripherally applied Rac1 inhibitor were devoid of a systemic component and did not come about via an alteration of basal mechanical and thermal sensitivity. These results underline the importance of Rac1 in the periphery in the modulation of nociceptive stimuli, in the context of both mechanical and thermal modalities.

MMP9 is under the transcriptional control of *Ets1* and belongs to a family of extracellular proteases called matrix metalloproteinases (MMPs) [51]. MMPs play a critical role in neuroinflammation through the cleavage of the extracellular matrix (ECM) proteins, cytokines, and chemokines [52-54]. Consistent with previously reported changes in MMP9 expression in nervous tissue following damage to peripheral nerves [33,35,52,55,56], we observed a 5-fold increase in MMP9 expression following GMCSF stimulus in sensory neurons. Intrathecally administered MMP9 inhibitors have been shown to block mechanical allodynia associated with peripheral inflammation [36] as well as nerve injury [35]. Functional experiments performed in the current study show that MMP9 inhibition in the periphery leads to significant protection from GMCSF-mediated mechanical hypersensitivity in the absence of a systemic influence or without affecting basal nociception. Interestingly, these findings thus indicate that MMP9 exerts pronociceptive effects not only in the central terminals but also at the peripheral nerve endings. Our findings further suggest that MMP9 may be linked to the role of GMCSF in tumor-associated pain, since both inflammatory and neuropathic mechanisms contribute to cancer pain. However, peripheral blockade of MMP9 did not affect GMCSF-mediated thermal hyperalgesia, which is consistent with a lack of its role in inflammatory thermal hyperalgesia.

Calpains are a family of ubiquitously expressed calcium-dependent cysteine proteases and have been implicated in several cellular processes such as proliferation, apoptosis and differentiation [57-59], pathological conditions including neuronal plasticity [60], neuronal cell death [61]. Proteolytic targets of calpain include cytoskeletal proteins such as neurofilament, spectrin, and membrane proteins such as growth factor receptors [62,63], cytokines [64,65] and transcription factors [66]. In the current study, long-term GMCSF or GCSF exposure led to robust transcriptional increase in the expression of calpain 2 and 7, but not other calpain family members, in sensory neurons. However, inhibiting the calpain 2 protease by using a specific calpain I/II inhibitor in the periphery did not have any impact on modulating GMCSF-induced mechanical and thermal hyperalgesia. This lack of phenotypic changes is not due to a lack of efficacy of the inhibitor since intrathecal application of comparable concentrations has been shown to significantly reduce zymosan-mediated thermal hypersensitivity in rats [37] or lysophosphatidic acid (LPA)-mediated thermal hyperalgesia in mice [38]. Thus, our results indicate that calpain is dispensable for GMCSF-induced nociceptive sensitization in the periphery; however, they do not rule out that GMCSF-mediated induction of calpain expression may be modulating other functions and processes in the DRG which were not studied here.

TNFα is a proinflammatory chemokine which was previously studied intensively in the context of nociceptive modulation [67,68]. Importantly, intraperitoneal application of TNFα decoy receptor etanercept relieved mice from tumor-mediated hyperalgesia [42]. However, it was also reported that intrathecally and intraperitoneally applied etanercept protects the mice from diabetic neuropathy-induced mechanical hyperalgesia but intraplantar application of the same inhibitor showed inefficient in protecting the mice from diabetic neuropathy-induced hypersensitivity [40]. Consistent with this observation, we observed that peripheral inhibition of TNFα is not sufficient to abrogate the nociceptive stimulus-mediated sensitivity. Taken together, these observations suggest that TNFα is recruited downstream of other tumor-associated mediators.

Other than the genes directly regulated by G-/GMCSF transcriptionally, our systems level network analysis revealed several other pathways which could be directly connected to the G-/GMCSF-mediated genes, such as IKK-NF-κB pathway. This classical, canonical pathway involves TNFα or IL-1β stimuli via respective receptors leading to the activation of an inhibitor of the NF-κB (I-κB) kinase complex, consisting of the regulatory subunit I-κB kinases (IKKs). This pathway is crucial for the activation of innate immunity and inflammation [69]. Functionally, increased NF-κB levels in DRG neurons following sciatic nerve crush [70] or upregulation of NF- κB transactivation following sciatic nerve transection [71] have been reported. Pharmacological intervention at several nodes of the NF-κB pathway has been shown to modulate nociceptive responses [69]. Inactivation of NF-κB specifically in primary sensory neurons of DRG via Cre-LoxP-mediated deletion of IKKB inhibitor kappa B kinase beta (IKKβ) has been shown to have a protective effect on nerve injury-mediated hyperalgesia [72]. Interestingly, the expression of TNFα and IL-1β robustly increased following G-/GMCSF stimuli in the current study, implying a clearer activation of NF-κB pathway in sensory neurons. Another molecular pathway which emerged to be very closely connected to G-/GMCSF-mediated transcriptome in sensory neurons is caspase signaling. Caspases are a family of proteases and play an important role in mediating programmed cell death following different noxious stimuli [73,74]. Peripheral nerve injury promotes neuronal cell death in the spinal dorsal horn [75-77] and arresting this nerve injury-induced neuronal loss in the spinal dorsal horn by blocking caspase activity reduces neuropathy-induced pain sensitivity [78]. Caspase signaling pathways differentially contribute to neuropathy-induced and TNF-mediated pain behaviors [79]. Our results on network analysis indicate that GMCSF signaling may be interlinked with TNF-alpha and caspase signaling in DRG neurons.

Thus, the striking changes we report in the transcription of many pain-related ion channels, chemokines, growth factors and proteases among several other classes of genes in DRG neurons following prolonged exposure to G-/GMCSF imply that G-/GMCSF signaling is a trigger point for activation of multiple pain modulatory pathways and that blocking the G-/GMCSF signaling may be very effective in alleviating a broad set of pain disorders.

## Conclusion

In summary, the current study demonstrates genome-wide transcriptome changes following chronic G-/GMCSF stimulus in the sensory neurons. Utilizing state-of-the art *in silico* systems level analysis, this study not only reveals that several key pain-related genes to be transcriptional targets of G-/GMCSF signaling, but also provides novel insights into network interactions with several other novel candidate genes. Using *in vivo* pharmacology, we provide the importance of peripheral MMP9 and Rac1 signaling in inhibiting GMCSF-mediated mechanical and thermal hypersensitivity. Thus, with integrative approach of genomics, bioinformatics, *in vivo* pharmacology and behavioral analyses, this study advances the understanding of nociceptive mechanisms in sensory neurons and provides a basis for further pursuing G-/GMCSF signaling in therapeutic treatment of pain disorders.

## Abbreviations

DRG: Dorsal root ganglion; GMCSF: Granulocyte macrophage colony stimulating factor; GCSF: Granulocyte colony stimulating factor; PBS: Phosphate buffered saline.

## Competing interests

The authors declare that they have no competing interests.

## Authors’ contributions

KB performed the major portion of the experiments, analyzed data and wrote the manuscript. VV and PG contributed to experiments and performed data analysis. VPS and RS contributed to the bioinformatics part of the study. RK designed and supervised the study and wrote the manuscript. All authors read and approved the final manuscript.

## Supplementary Material

Additional file 1: Table S1 Significantly regulated genes following GMCSF or GCSF chronic stimulus in the sensory neurons with the 2 fold cut-off and pBH < 0.05 (two-tailed *t*-test assuming equal variance, P with Benjamini and Hochberg False Discovery Rate correction <0.05). Click here for file

Additional file 2: Figure S1 Symbols used to represent different functional classes of protein to represent direct-interactions networks in Figures 3 and 4 and network interactions in Additional file 3: Figure S2 and Additional file 4: Figure S3. Click here for file

Additional file 3: Figure S2 Direct-interactions-network analysis on the gene pool commonly up- and down-regulated following GMCSF or GCSF exposure in the sensory neurons. Gene pool with fold-change between +4 and −4 as compared to control-treated sensory neurons and P-BH < 0.05 (*t*-test, P with Benjamini and Hochberg False Discovery Rate < 0.05). Genes upregulated and downregulated in GMCSF-dependent manner are marked with red and blue circles respectively. Please see Additional file 2: Figure S1 for information on legends. Click here for file

Additional file 4: Figure S3 Gene-ontology enrichment analysis on gene pools induced by GMCSF **(A)** and GCSF **(B)**. Analysis was performed using bioCompendium online repository (http://biocompendium.embl.de) using the gene pool with fold-change between +2 and −2, as compared to control-treated sensory neurons and P-BH < 0.05 (*t*-test, P with Benjamini and Hochberg False Discovery Rate <0.05). Percent of G-/GM-CSF induced genes over total input number of genes significantly enriched are represented in both panels (P-BH < 0.05, as compared to mouse genome, hypergeometric statistical method). Click here for file

Additional file 5: Figure S4 Top scored network obtained from the gene pool regulated by GM-CSF stimulus in sensory neurons, using the same set of genes explained in the Figure 1-A. Genes upregulated and downregulated in GMCSF-dependent manner are shown with red and blue circles respectively. Thick cyan lines indicate the fragments of canonical pathways. Please refer Additional file 2: Figure S1 for the symbols representing different generic classes of proteins. Click here for file

Additional file 6: Table S2 Impact of inhibition of Rac1, MMP9, Calpain2 or TNFα on GMCSF-mediated mechanical hypersensitivity. Response frequencies to calibrated von Frey filaments of different strength at paws ipsilateral and contralateral to intraplantar GMCSF administration are shown as compared to corresponding vehicle-treated mice. * denotes P ≤ 0.05 as compared to basal values, ^†^ denotes P ≤ 0.05 relative to corresponding vehicle-treated group, One-Way ANOVA with repeated measures followed Fisher’s LSD Post-hoc analysis , n = 6 mice per group. Click here for file

Additional file 7: Table S3 Impact of Inhibition of Rac1, MMP9, Calpain2 or TNFα on GMCSF-mediated thermal hypersensitivity. Withdrawal latency in seconds to calibrated radiant heat from the paws ipsilateral and contralateral to intraplantar GMCSF administration are shown as compared to corresponding vehicle-treated mice. * denotes P ≤ 0.05 as compared to basal values, ^†^ denotes P ≤ 0.05 relative to corresponding vehicle treated group, One-Way ANOVA with repeated measures followed Fisher’s LSD Post-hoc analysis, n = 6 mice per group. Click here for file

Additional file 8: Figure S5 Systemic effects of intraplantar application of inhibitors of specific pathways on GMCSF-induced nociceptive sensitization. Changes in the GMCSF-mediated mechanical hypersensitivity in the paw ipsilateral to the GMCSF-injected paw following Rac1 **(A)** or MMP9 **(B)** inhibitors application in the paw contralaeral to GMCSF-injected paw or changes in the GM-CSF-mediated mechanical hypersensitivity in the paw contralateral to the GMCSF-injected paw following Rac1 **(E)** or MMP9 **(F)** inhibitors application in the paw contralaeral to GMCSF-injected paw as compared to corresponding vehicle-treated mice are shown. Response frequency to the von Frey filament at 0.16 g force is represented on the Y-axis. Changes in the GMCSF-mediated thermal hypersensitivity in the paw ipsilateral to the GMCSF-injected paw following Rac1 **(C)** or MMP9 **(D)** inhibitors or changes in the GMCSF-mediated thermal hypersensitivity in the paw contralateral to the GMCSF-injected paw following Rac1 **(G)** or MMP9 **(H)** inhibitors as compared to corresponding vehicle-treated group of mice. Withdrawal latency in seconds to calibrated radiant heat is represented. * denotes P ≤ 0.05 as compared to basal values, One-Way ANOVA with repeated measures followed Fisher’s LSD Post-hoc analysis, n = 6 mice per group. Click here for file

Additional file 9: Figure S6 Effect of blockade of Rac1 or MMP9 on basal mechanical and thermal sensitivity. Changes in the perception of mechanical sensitivity in response to von Frey filaments of increasing strength in the paw ipsilateral to the Rac1 **(A)** or MMP9 **(C)** inhibitor application is compared to the response frequency before inhibitor application (basal). Percentage Response frequency to the von Frey filament at 0.16 g force is represented on the Y-axis. Changes in the response to thermal stimuli following Rac1 **(B)** or MMP9 **(D)** inhibitors as compared to basal reading. Withdrawal latency in seconds to calibrated radiant heat is represented. * denotes P ≤ 0.05 as compared to basal values, One-Way ANOVA with repeated measures followed Fisher’s LSD Post-hoc analysis , n = 6 mice per group. Click here for file
