# Supplementary figures and images for: Transcriptional mechanisms underlying sensitization of peripheral sensory neurons by Granulocyte-/Granulocyte-macrophage colony stimulating factors
Source: Mol Pain. 2013 Sep 25;9:48. doi: 10.1186/1744-8069-9-48 (PMC3852053; doi:10.1186/1744-8069-9-48)

Suppl. Fig. 1

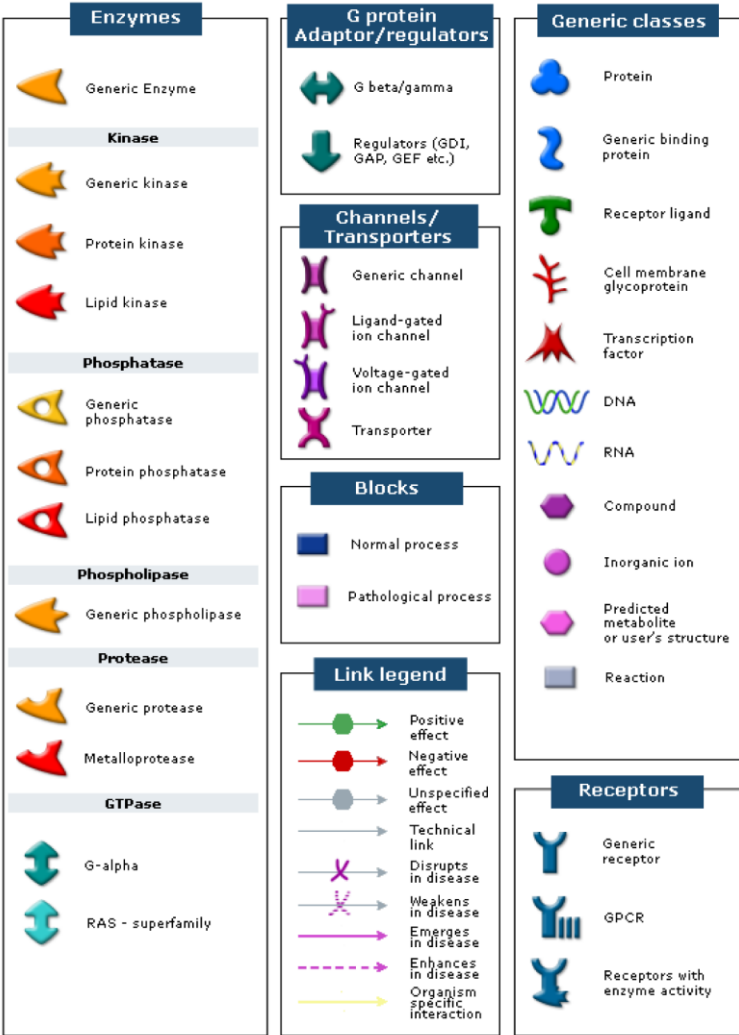

Supplement: Additional file 2: Figure S1 — Symbols used to represent different functional classes of protein to represent direct-interactions networks in Figures 3 and 4 and network interactions in Additional file 3: Figure S2 and Additional file 4: Figure S3. [file 1744-8069-9-48-S2.pdf]

Suppl. Fig. 2

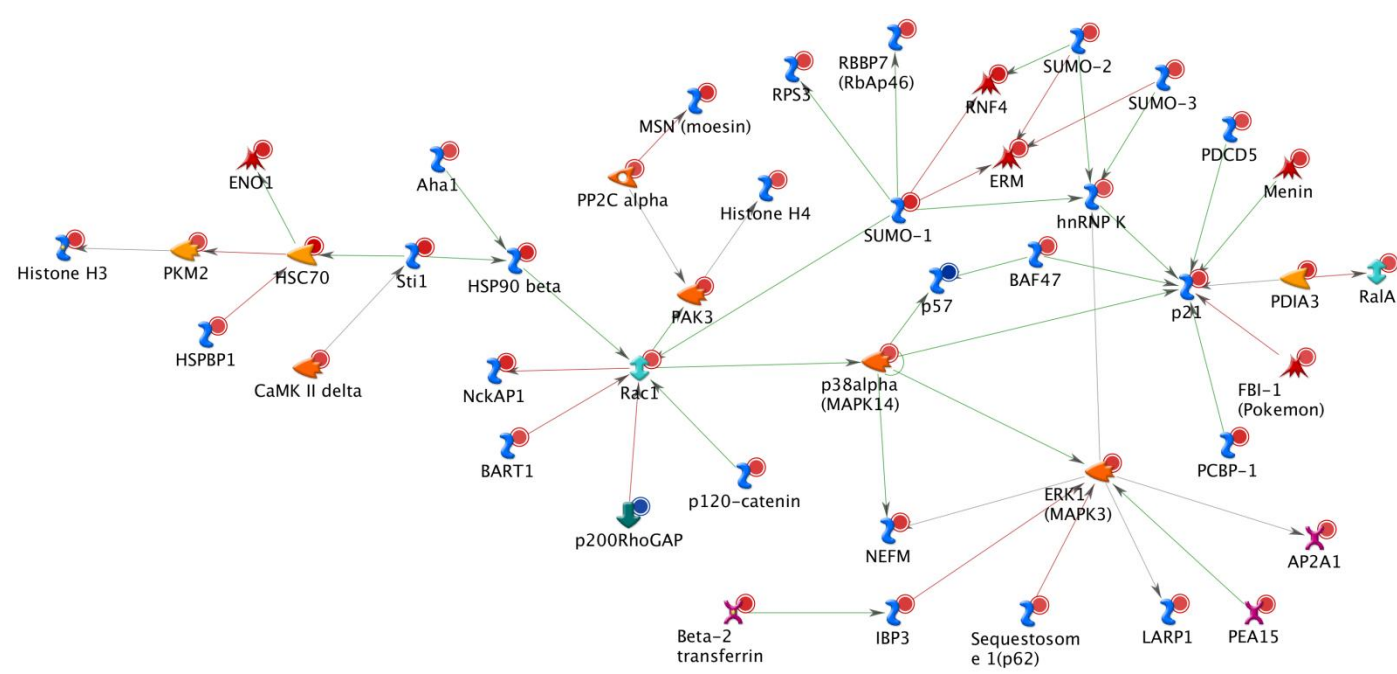

Supplement: Additional file 3: Figure S2 — Direct-interactions-network analysis on the gene pool commonly up- and down-regulated following GMCSF or GCSF exposure in the sensory neurons. Gene pool with fold-change between +4 and −4 as compared to control-treated sensory neurons and P-BH < 0.05 (t-test, P with Benjamini and Hochberg False Discovery Rate < 0.05). Genes upregulated and downregulated in GMCSF-dependent manner are marked with red and blue circles respectively. Please see Additional file 2: Figure S1 for information on legends. [file 1744-8069-9-48-S3.pdf]

Suppl. Fig. 3

(A) GMCSF-induced gene pool

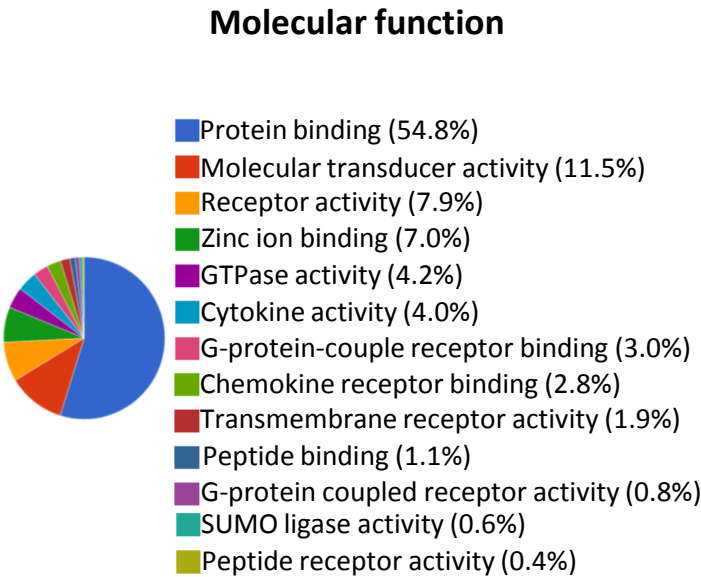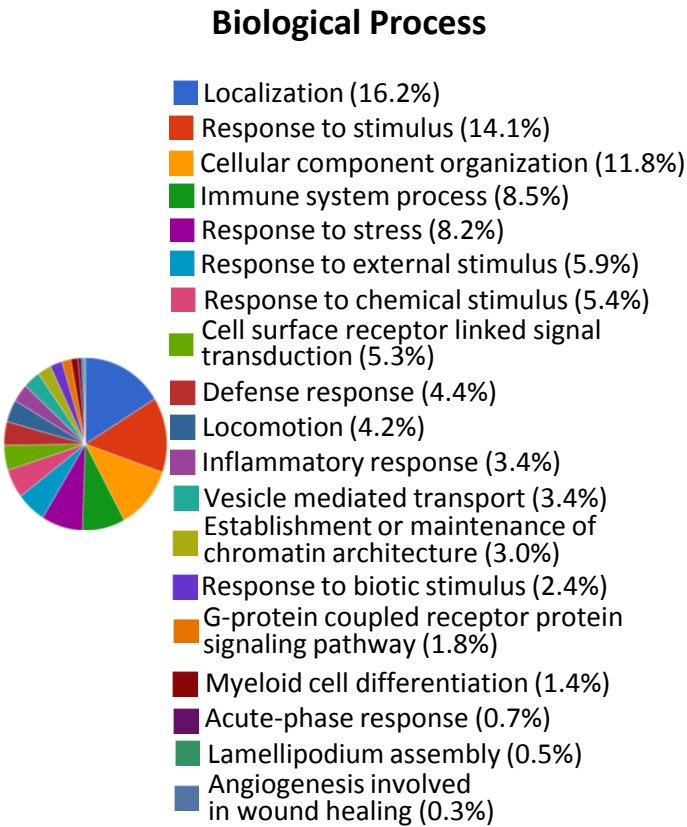

(B) GCSF-induced gene pool

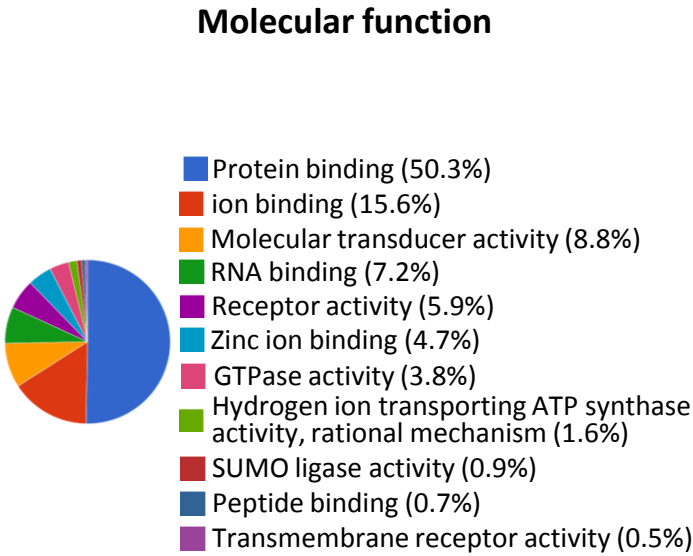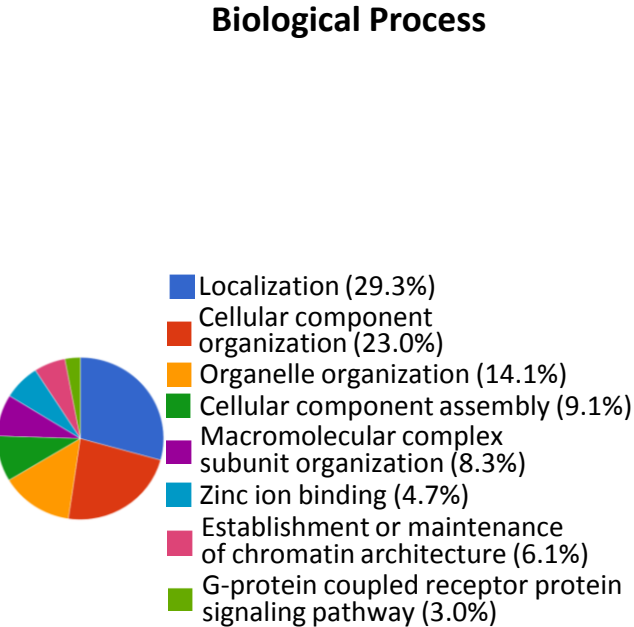

Supplement: Additional file 4: Figure S3 — Gene-ontology enrichment analysis on gene pools induced by GMCSF (A) and GCSF (B). Analysis was performed using bioCompendium online repository (http://biocompendium.embl.de) using the gene pool with fold-change between +2 and −2, as compared to control-treated sensory neurons and P-BH < 0.05 (t-test, P with Benjamini and Hochberg False Discovery Rate <0.05). Percent of G-/GM-CSF induced genes over total input number of genes significantly enriched are represented in both panels (P-BH < 0.05, as compared to mouse genome, hypergeometric statistical method). [file 1744-8069-9-48-S4.pdf]

### Suppl. Fig. 4

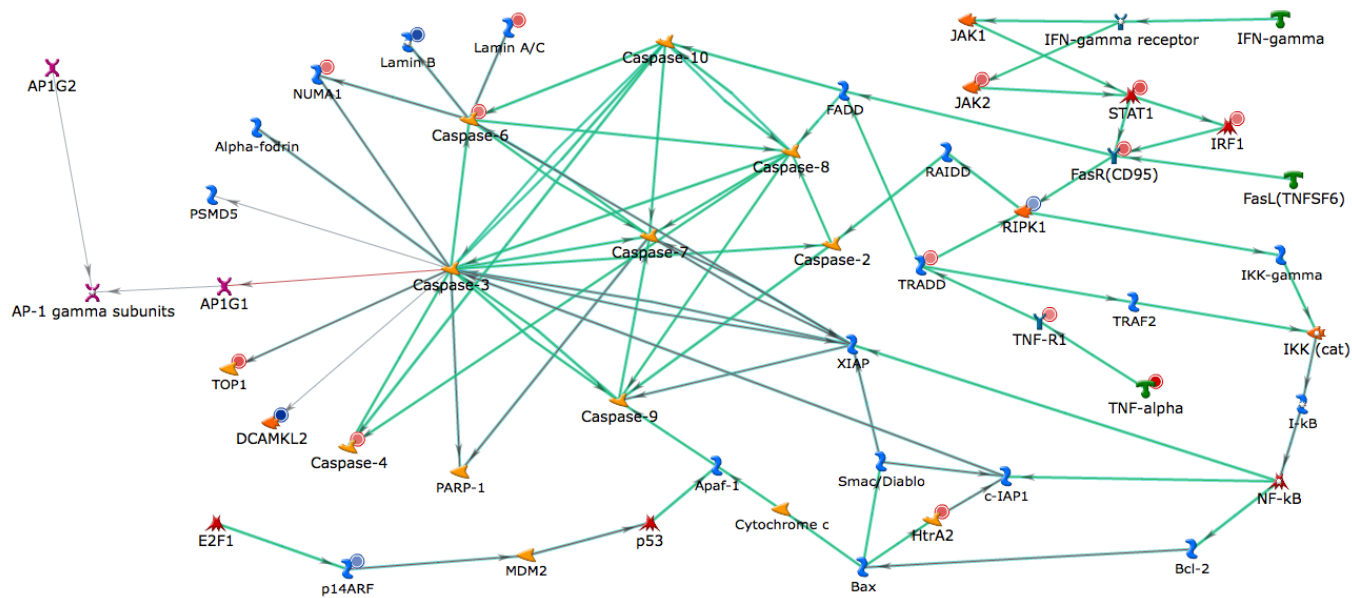

Supplement: Additional file 5: Figure S4 — Top scored network obtained from the gene pool regulated by GM-CSF stimulus in sensory neurons, using the same set of genes explained in the Figure 1-A. Genes upregulated and downregulated in GMCSF-dependent manner are shown with red and blue circles respectively. Thick cyan lines indicate the fragments of canonical pathways. Please refer Additional file 2: Figure S1 for the symbols representing different generic classes of proteins. [file 1744-8069-9-48-S5.pdf]

Suppl. Fig. 5

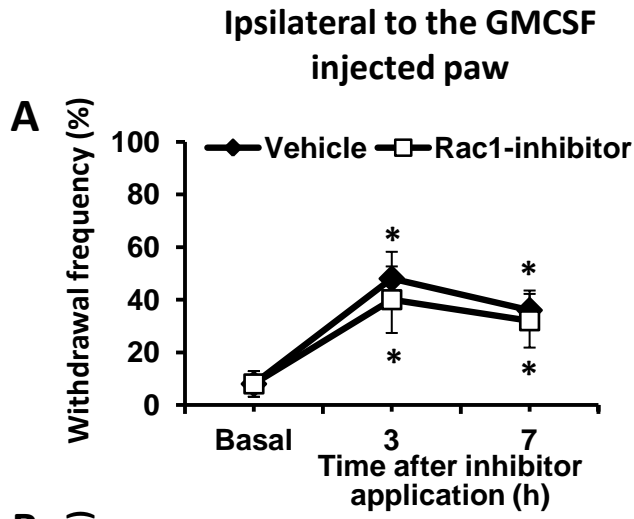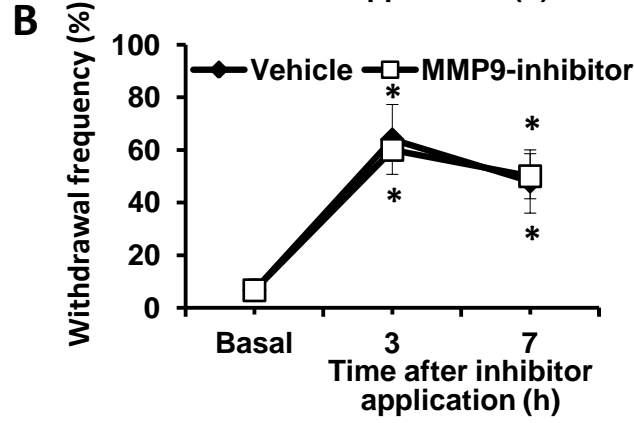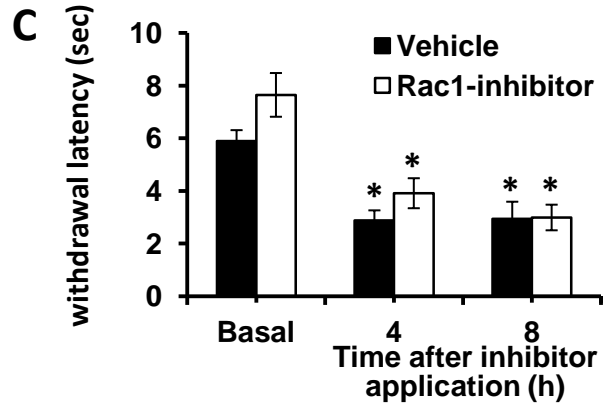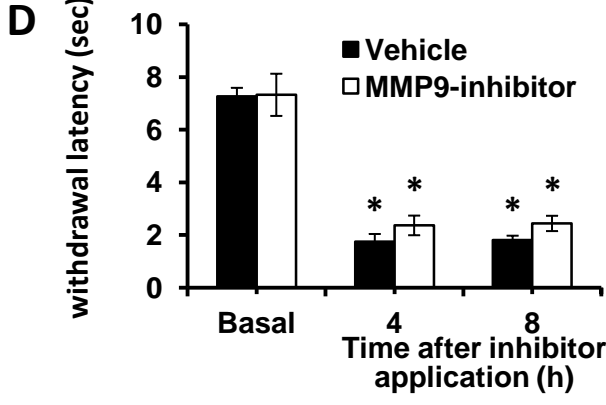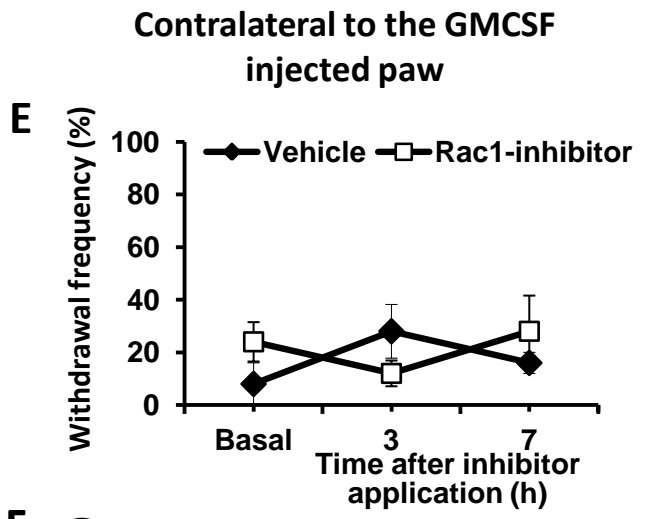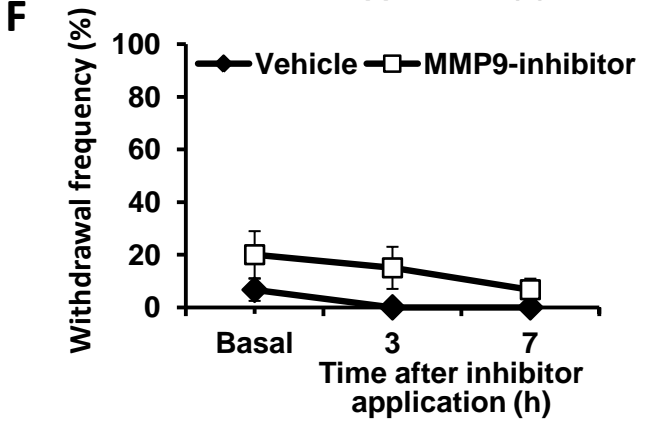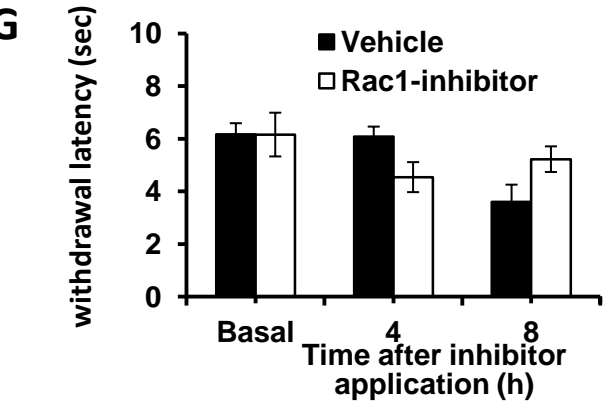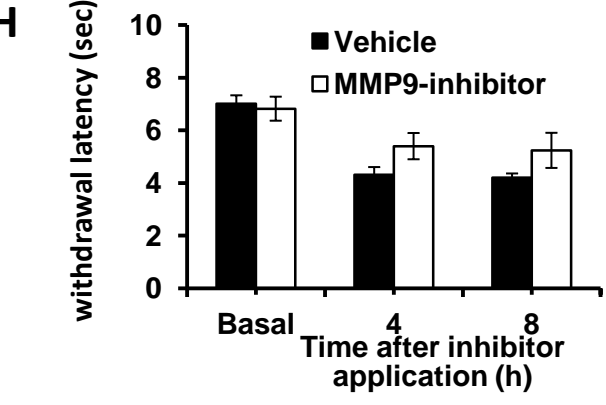

Supplement: Additional file 8: Figure S5 — Systemic effects of intraplantar application of inhibitors of specific pathways on GMCSF-induced nociceptive sensitization. Changes in the GMCSF-mediated mechanical hypersensitivity in the paw ipsilateral to the GMCSF-injected paw following Rac1 (A) or MMP9 (B) inhibitors application in the paw contralaeral to GMCSF-injected paw or changes in the GM-CSF-mediated mechanical hypersensitivity in the paw contralateral to the GMCSF-injected paw following Rac1 (E) or MMP9 (F) inhibitors application in the paw contralaeral to GMCSF-injected paw as compared to corresponding vehicle-treated mice are shown. Response frequency to the von Frey filament at 0.16 g force is represented on the Y-axis. Changes in the GMCSF-mediated thermal hypersensitivity in the paw ipsilateral to the GMCSF-injected paw following Rac1 (C) or MMP9 (D) inhibitors or changes in the GMCSF-mediated thermal hypersensitivity in the paw contralateral to the GMCSF-injected paw following Rac1 (G) or MMP9 (H) inhibitors as compared to corresponding vehicle-treated group of mice. Withdrawal latency in seconds to calibrated radiant heat is represented. * denotes P ≤ 0.05 as compared to basal values, One-Way ANOVA with repeated measures followed Fisher’s LSD Post-hoc analysis, n = 6 mice per group. [file 1744-8069-9-48-S8.pdf]

Suppl. Fig. 6

Mechanical Sensitivity

Heat Sensitivity

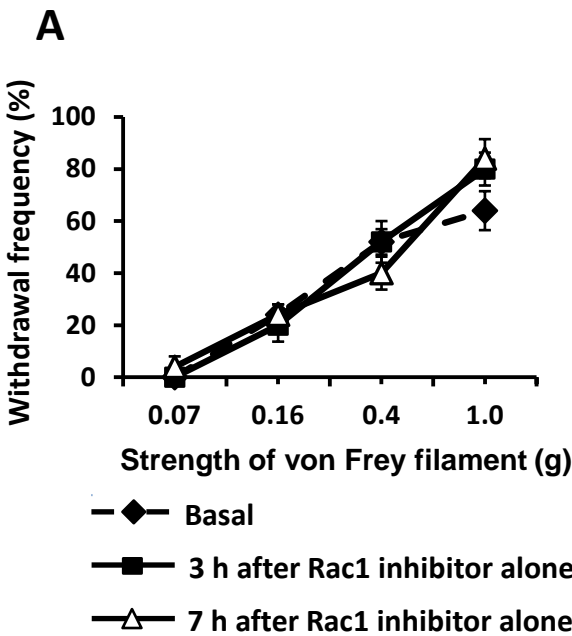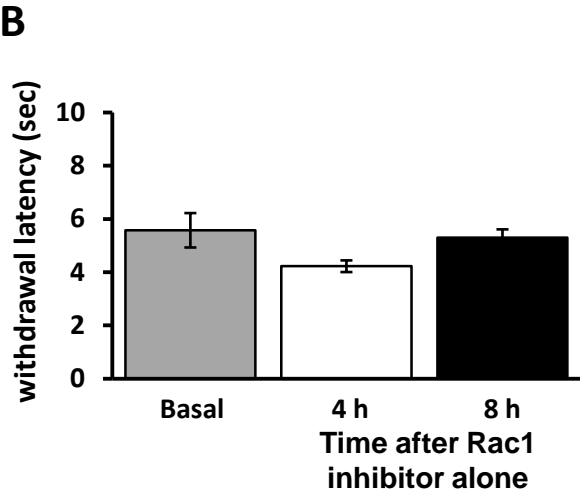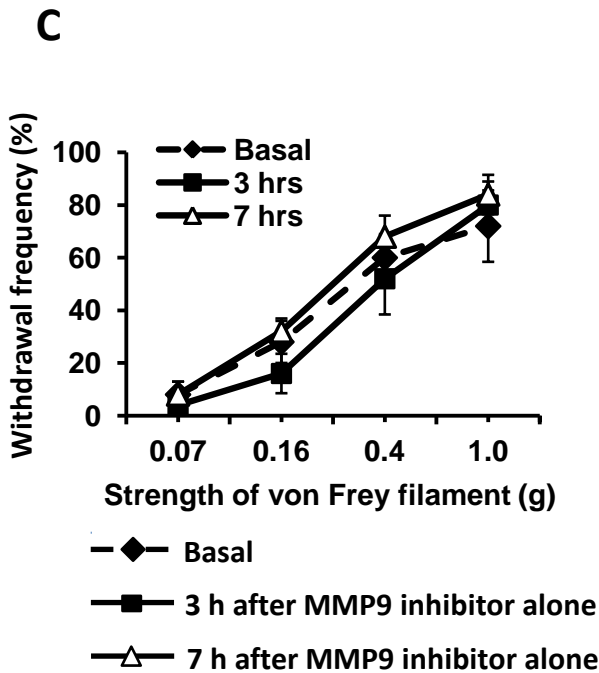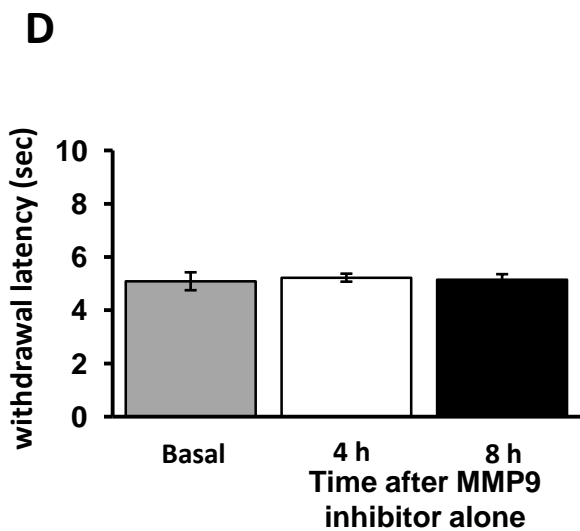

Supplement: Additional file 9: Figure S6 — Effect of blockade of Rac1 or MMP9 on basal mechanical and thermal sensitivity. Changes in the perception of mechanical sensitivity in response to von Frey filaments of increasing strength in the paw ipsilateral to the Rac1 (A) or MMP9 (C) inhibitor application is compared to the response frequency before inhibitor application (basal). Percentage Response frequency to the von Frey filament at 0.16 g force is represented on the Y-axis. Changes in the response to thermal stimuli following Rac1 (B) or MMP9 (D) inhibitors as compared to basal reading. Withdrawal latency in seconds to calibrated radiant heat is represented. * denotes P ≤ 0.05 as compared to basal values, One-Way ANOVA with repeated measures followed Fisher’s LSD Post-hoc analysis , n = 6 mice per group. [file 1744-8069-9-48-S9.pdf]
