# Supplementary material for: Transcriptional mechanisms underlying sensitization of peripheral sensory neurons by Granulocyte-/Granulocyte-macrophage colony stimulating factors
Source: Mol Pain. 2013 Sep 25;9:48. doi: 10.1186/1744-8069-9-48 (PMC3852053; doi:10.1186/1744-8069-9-48)
Supplement: Additional file 6: Table S2 — Impact of inhibition of Rac1, MMP9, Calpain2 or TNFα on GMCSF-mediated mechanical hypersensitivity. Response frequencies to calibrated von Frey filaments of different strength at paws ipsilateral and contralateral to intraplantar GMCSF administration are shown as compared to corresponding vehicle-treated mice. * denotes P ≤ 0.05 as compared to basal values, † denotes P ≤ 0.05 relative to corresponding vehicle-treated group, One-Way ANOVA with repeated measures followed Fisher’s LSD Post-hoc analysis , n = 6 mice per group. [file 1744-8069-9-48-S6.docx]

**Suppl.Table 2**

| **Von Frey filament (g)** | **Percentage of withdrawal frequency (±SEM)** | | | | | | | | | | | |
| --- | --- | --- | --- | --- | --- | --- | --- | --- | --- | --- | --- | --- |
|  | **Paw ipsilateral to GMCSF + inhibitor application** | | | | | | **Paw contralateral to GMCSF + inhibitor application** | | | | | |
|  | **Basal** | | **3 h** | | **7 h** | | **Basal** | | **3 h** | | **7 h** | |
|  | **Veh** | **Inh** | **Veh** | **Inh** | **Veh** | **Inh** | **Veh** | **Inh** | **Veh** | **Inh** | **Veh** | **Inh** |
| **Rac1 inhibition** | | | | | | | | | | | | |
| **0.07** | 4.0 (4) | 12.0 (4) | 56.6 (8)* | 23.3 (8) † | 33.3 (6)* | 6.6 (4) † | 8.0 (4) | 8.0 (8) | 23.3 (6) | 20 (7) | 13.3 (6) | 0 |
| **0.16** | 12.0 (4) | 8.0 (4) | 50.0 (6)* | 26.6 (11)† | 46.6 (8)* | 23.3 (6) † | 24.0 (11) | 16.0 (11) | 10.0 (6) | 30.0 (10) | 16.6 (10) | 26.6 (6) |
| **0.4** | 20.0 (6) | 4.0 (4) | 73.3 (8)* | 40.0 (10)* † | 53.3 (4)* | 33.3 (9)* † | 32.0 (13) | 12.0 (8) | 40.0 (7) | 43.3 (13) | 26.6 (6) | 30.0 (8) |
| **1.0** | 52.0 (10) | 40.0 (14) | 86.6 (6*) | 66.6 (4) | 76.6 (9) | 53.3 (12) | 52.0 (12) | 56.0 (13) | 40.0 (7) | 60.0 (11) | 46.6 (9) | 56.6 (14) |
| **MMP-9 inhibition** | | | | | | | | | | | | |
| **0.07** | 3.3 (3) | 3.3 (3) | 36.7 (10)* | 40.0 (10)* † | 36.7 (9)* | 16.7 (6)* † | 0 | 3.3 (3) | 10.0 (4) | 16.7 (6) | 13.3 (10) | 16.7 (6) |
| **0.16** | 6.7 (4) | 6.6 (4) | 76.7 (9)* | 56.7 (6) † | 80.0 (5)* | 73.3 (6)* † | 6.7 (4) | 20.0 (9) | 36.7 (8) | 26.7 (10) | 20.0 (7) | 23.3 (6) |
| **0.4** | 26.7 (6) | 26.7 (4) | 76.7 (9)* | 56.7 (8)* | 63.3 (12)* | 40.0 (5) | 23.3 (8) | 33.3 (7) | 43.3 (8) | 56.7 (3) | 43.3 (10) | 43.3 (8) |
| **1.0** | 46.7 (10) | 46.7 (8) | 76.7 (3)* | 56.7 (6) | 80.0 (5)* | 73.3 (7)* | 46.7 (8) | 46.7 (4) | 46.7 (8) | 56.7 (6) | 43.3 (14) | 53.3 (11) |
| **Calpain-2 inhibition** | | | | | | | | | | | | |
| **0.07** | 3.3 (3) | 3.3 (3) | 72.0 (8)* | 60.0 (11)* | 60.0 (14)* | 64.0 (10)* | 0 | 0 | 0 | 8 (5) | 16 (7) | 8 (5) |
| **0.16** | 26.7 (10) | 13.3 (7) | 72.0 (5)* | 96.0 (4)* | 68.0 (14)* | 88.0 (8)* | 16.7 (6) | 10.0 (4) | 0 | 12.0 (8) | 20 (15) | 32.0 (10) |
| **0.4** | 50.0 (10) | 33.3 (12) | 76.0 (4)* | 80.0 (11)* | 84.0 (7)* | 80.0 (6)* | 36.7 (6) | 33.3 (10) | 16.0 (7) | 32.0 (8) | 24.0 (4) | 4.0 (4) |
| **1.0** | 60.0 (9) | 53.3 (8) | 84.0 (4)* | 96.0 (4)* | 96.0 (4)* | 84.0 (7)* | 46.7 (10) | 40.0 (7) | 40.0 (11) | 44.0 (16) | 44.0 (10) | 32.0 (8) |
| **TNF-alpha inhibition** | | | | | | | | | | | | |
| **0.07** | 4.0 (4) | 12.0 (8) | 32.0 (8)* | 48.0 (13.5)* | 0 | 0 | 4.0 (4) | 20.0 (6.3 | 4.0 (4) | 12.0 (5) | 0 | 0 |
| **0.16** | 12.0 (5) | 8.0 (5) | 60.0 (6)* | 72.0 (8)* | 48.0 (5)* | 36.0 (7)* | 36.0 (12) | 32.0 (8) | 8.0 (5) | 20.0 (15) | 8.0 (5) | 8.0 (5) |
| **0.4** | 12.0 (8) | 16.0 (7) | 80.0 (0)* | 72.0 (8)* | 68.0 (5)* | 60.0 (6)* | 12.0 (8) | 20.0 (6) | 40.0 (6) | 40.0 (6) | 40.0 (6) | 24.0 (7) |
| **1.0** | 32.0 (17) | 24.0 (4) | 72.0 (8) | 80.0 (6) | 84.0 (4) | 76.0 (12) | 44.0 (15 ) | 36.0 (18) | 40.0 (6) | 60.0 (6) | 44.0 (4) | 44.0 (12) |
