# Supplementary material for: Transcriptional mechanisms underlying sensitization of peripheral sensory neurons by Granulocyte-/Granulocyte-macrophage colony stimulating factors
Source: Mol Pain. 2013 Sep 25;9:48. doi: 10.1186/1744-8069-9-48 (PMC3852053; doi:10.1186/1744-8069-9-48)
Supplement: Additional file 7: Table S3 — Impact of Inhibition of Rac1, MMP9, Calpain2 or TNFα on GMCSF-mediated thermal hypersensitivity. Withdrawal latency in seconds to calibrated radiant heat from the paws ipsilateral and contralateral to intraplantar GMCSF administration are shown as compared to corresponding vehicle-treated mice. * denotes P ≤ 0.05 as compared to basal values, † denotes P ≤ 0.05 relative to corresponding vehicle treated group, One-Way ANOVA with repeated measures followed Fisher’s LSD Post-hoc analysis, n = 6 mice per group. [file 1744-8069-9-48-S7.docx]

| **Thermal withdrawal latency (Sec)** | | | | | | | | | | | |  |  |
| --- | --- | --- | --- | --- | --- | --- | --- | --- | --- | --- | --- | --- | --- |
| **Paw ipsilateral to GMCSF + inhibitor application** | | | | | | **Paw contralateral to GMCSF + inhibitor application** | | | | | |  |  |
| **Basal** | | **4 h** | | **8 h** | | **Basal** | | **4 h** | | **8 h** | |  |  |
| **Veh** | **Inh** | **Veh** | **Inh** | **Veh** | **Inh** | **Veh** | **Inh** | **Veh** | **Inh** | **Veh** | **Inh** |  |  |
| **Rac1 inhibition** | | | | | | | | | | | | | |
| 5.2 (1.3) | 5.3 (0.9) | 2.1 (0.4) * | 4.3 (0.6) † | 1.8 (0.3) * | 2.9 (0.1) * † | 5.5 (0.8) | 5.5 (0.4) | 4.8 (0.7) | 5.4 (0.4) | 6.1 (0.7) | 4.7 (0.4) | | |
| **MMP-9 inhibition** | | | | | | | | | | | | | |
| 5.6 (0.6) | 5.5 (0.7) | 3.7 (0.6) * | 5.3 (0.8) | 3.2 (0.7) * | 3.5 (0.2) * | 4.6 (0.4) | 5.2 (0.4) | 6.2 (0.9) | 4.5 (0.2) | 5.5 (0.4) | 4.1 (0.4) | | |
| **Calpain-2 inhibition** | | | | | | | | | | | | | |
| 4.2 (0.4) | 3.6 (0.7) | 2.1 (0.6) * | 2.3 (0.7) * | 1.6 (0.3) * | 3.0 (0.7) | 4.3 (0.7) | 3.6 (0.4) | 3.8 (0.7) | 4.0 (0.6) | 4.2 (0.2) | 4.6 (0.9) | |  |
| **TNF-alpha inhibition** | | | | | | | | | | | | | |
| 7.3 (0.3) | 7.5 (1) | 3.3 (0.2) * | 2.8 (0.3) * | 2.5 (0.3) * | 3.4 (0.2) * | 7.0 (0.9) | 6.3 (0.5) | 7.4 (0.4) | 6.7 (0.4) | 6.7 (0.5) | 7.1 (0.6) | |  |

**Suppl. Table 3**
